# Inducible Nitric Oxide Synthase Promoter Haplotypes and Residential Traffic-Related Air Pollution Jointly Influence Exhaled Nitric Oxide Level in Children

**DOI:** 10.1371/journal.pone.0145363

**Published:** 2015-12-29

**Authors:** Muhammad T. Salam, Pi-Chu Lin, Sandrah P. Eckel, W. James Gauderman, Frank D. Gilliland

**Affiliations:** 1 Department of Preventive Medicine, University of Southern California Keck School of Medicine, Los Angeles, California, United States of America; 2 Department of Psychiatry, Kern Medical Center, Bakersfield, California, United States of America; 3 Molecular Technical Operations, Siemens HealthCare Diagnostics, East Walpole, Massachusetts, United States of America; Boston University, UNITED STATES

## Abstract

**Background:**

Exhaled nitric oxide (FeNO), a biomarker of airway inflammation, predicts asthma risk in children. We previously found that the promoter haplotypes in inducible nitric oxide synthase (*NOS2*) and exposure to residential traffic independently influence FeNO level. Because *NOS2* is inducible by environmental exposures such as traffic-related exposure, we tested the hypothesis that common *NOS2* promoter haplotypes modulate the relationship between residential traffic-related exposure and FeNO level in children.

**Methods:**

In a cross-sectional population-based study, subjects (N = 2,457; 7–11 year-old) were Hispanic and non-Hispanic white children who participated in the Southern California Children’s Health Study and had FeNO measurements. For residential traffic, lengths of local roads within circular buffers (50m, 100m and 200m radii around homes) around the subjects’ homes were estimated using geographic information system (GIS) methods. We interrogated the two most common *NOS2* promoter haplotypes that were found to affect FeNO level.

**Results:**

The relationship between local road lengths within 100m and 200m circular buffers and FeNO level varied significantly by one of the *NOS2* promoter haplotypes (P-values for interaction between road length and *NOS2* promoter haplotype = 0.02 and 0.03, respectively). In children who had ≤250m of local road lengths within 100m buffer around their homes, those with two copies of the haplotype had significantly lower FeNO (adjusted geometric mean = 11.74ppb; 95% confidence intervals (CI): 9.99 to 13.80) than those with no copies (adjusted geometric mean = 15.28ppb; 95% CI: 14.04 to 16.63) with statistically significant trend of lower FeNO level with increasing number of haplotype copy (P-value for trend = 0.002). In contrast, among children who had >250m of local road lengths within 100m buffer, FeNO level did not significantly differ by the haplotype copy-number (P-value for trend = 0.34). Similar interactive effects of this haplotype and local road lengths within 200m buffer on FeNO were also observed.

**Conclusions:**

Higher exposure from residential traffic nullifies the protective effect of one common *NOS2* promoter haplotype on FeNO level. Regulation of traffic-related pollution may protect children’s respiratory health.

## Introduction

The fractional concentration of nitric oxide (NO) in exhaled air (FeNO) is a noninvasive biomarker of aspects of airway inflammation [1–3]. In several epidemiological studies, FeNO level has been found to predict new-onset asthma and wheeze risk in children [4–6]. Recent studies also indicate that FeNO level provides independent information about airway inflammation in nearly 50% children with asthma, that is not reflected by sputum or blood eosinophilia [7, 8].

Nitric oxide (NO) plays an important role in regulating airway smooth muscle tone and is involved in a variety of physiological and pathological processes [9]. NO is generated endogenously from L-arginine by NO synthase (NOS). Two constitutive (encoded by *NOS1* and *NOS3*) and one inducible isoforms of NOS (iNOS, encoded by *NOS2*) are the sources of airway epithelium-derived NO [10]; nevertheless, FeNO appears to be predominantly determined by *NOS2* [11].

Atopic conditions (asthma and allergy), genetic, and environmental exposures are some of the major determinants of FeNO level. We and others have found that children with asthma and allergic rhinitis have significantly higher FeNO than children without these conditions [12–14]. Using data from the Southern California Children's Health Study (CHS), we previously reported that the two most common promoter haplotypes in *NOS2* significantly affects FeNO level in children [15], and were also associated with asthma risk and lung function growth in children [16].

Traffic exhaust is considered to be the one of the main sources of environmental pollution in urban areas. Higher FeNO has been attributed to urban air pollution [12, 17, 18]. Reactive oxygen species (ROS) and free radicals generated by traffic exhaust have adverse health effect on the respiratory system in children [19, 20]. We and others have found that length of roads around the home (a marker for residential traffic-related air pollution) was associated with higher FeNO in children [12, 21, 22].

Because *NOS2* is highly inducible by environmental exposures, we hypothesized that 1) common *NOS2* promoter haplotypes influence the relationship between road length around homes and FeNO and 2) the joint effects of *NOS2* promoter haplotypes and length of road traffic exposure on FeNO vary by asthma status in children.

## Materials and Methods

### Study design and subjects

The subjects were kindergarten and first grade (5–8 years old at cohort entry) school children who were recruited in a new cohort of the Children’s Health Study in 2003 from 12 communities of southern California. The detailed study design and subject recruitment have been described earlier [12, 23]. Briefly, parents or guardians of study participants completed the written informed consent and questionnaires, and the study subjects assented. The University of Southern California Institutional Review Board approved the study protocol. In 2005–2006, FeNO measurements were collected from 2,948 children (7–11 years old). Because genotyping was done for Hispanic and non-Hispanic white children, we excluded 433 children who belonged to other racial/ethnic groups (e.g., Asian, African-American and mixed). Of the 2515 Hispanic and non-Hispanic white children with FeNO and genotypic data, local length of road measures was unavailable for 58 children. Therefore, a total of 2457 children were available for the final analysis.

### FeNO measurement

Details of FeNO collection, quality control, and data transformation have been described earlier [24, 25]. FeNO was collected from children at school during October to June in 2005–2006 using an offline (100 ml/sec flow) technique according to the American Thoracic Society guidelines [26]. In a separate study performed in a subset group (N = 361) of participants, offline FeNO measurements were validated by an online (50 ml/sec flow) technique and the quality control measures and study findings were previously published [25]. In that paper, we reported that online FeNO could be predicted quite reliably from offline FeNO (adjusted R^2^ = 0.94) when appropriate adjustments for ambient NO and the time spent between collection and work-up for FeNO measurement was done in a regression model [25]. Offline FeNO data were transformed to predicted online FeNO data for the analyses in this paper.

### Assessment of traffic-related exposure

Because earlier work showed that total local road lengths within 50m, 100m and 200m buffers around subjects' home were associated with higher FeNO levels in children [12, 21, 22], we selected these exposures to evaluate their joint effects with the two most common *NOS2* promoter haplotypes on FeNO levels in children. Based on the residential addresses at the time of FeNO measurement (2005–2006), the road lengths within these circular buffers around each participant’s was calculated based on TeleAtlas Multi-Net road class data (TeleAtlas 2002). Data of length of local roads obtained from major or minor collectors were equivalent to functional road class (FRC) 5 or FRC-6, respectively [12].

### Selection of *NOS2* promoter haplotypes

The criteria for haplotype-tagged single nucleotide polymorphism (htSNPs) selection and genotyping methods have been described in detail previously [15]. Seven SNPs (rs4795080, rs2779253, rs1889022, rs10853181, rs2531866, rs1014025, and rs2531872) were included to estimate the haplotype in *NOS2* promoter region. These htSNPs had minor allele frequency ≥0.05 and were able to explained >90% of the haplotype diversity of the *NOS2* promoter region. Haplotypes for each racial/ethnic group were estimated using the TagSNPs program (http://www-rcf.usc.edu/~stram/tagSNPs.html). We used 233 ancestry informative markers (AIMs) to determine genetic ancestry and account for population stratification. Genotyping was done using the Illumina BeadArray platform. All SNPs had call rates greater than 99%. We used the STRUCTURE program (available at http://pritch.bsd.uchicago.edu/structure.html) to differentiate the four major ancestral populations. More information on the basic algorithm of the program and methods utilized in similar multiethnic populations has been published elsewhere [27–29].

### Assessment of covariates

Information of race/ethnicity, physician-diagnosed asthma, history of respiratory allergy (allergic rhinitis and/or hay fever), asthma medication use during the past 12 months, annual family income, parental education and exposure to secondhand tobacco smoke were based on parental reports. Height and weight were measured on the day of FeNO testing. Age- and sex-specific body mass index (BMI) percentiles was computed using the Centers for Disease Control and Prevention body mass index growth charts (http://www.cdc.gov/NCCDPHP/dnpa/growthcharts/resources/sas.htm) and categories of BMI (e.g., underweight, normal, overweight and obese) were defined.

### Statistical analysis

Predicted online FeNO was natural-log-transformed to adhere to the assumptions of multiple linear regression models because the FeNO distribution was right-skewed. The road length measures were centered at their respective means and were scaled to 100m, 300m, and 1000m for total length of roads in 50m, 100m, and 200m buffers, respectively. Descriptive analyses were performed to describe characteristics of the study population. Spearman correlations were used for pairwise correlations of *NOS2* promoter haplotypes and road length measures. Linear regression models with appropriate interaction terms were used to examine the joint effects of *NOS2* promoter haplotypes and road length measures on FeNO level using likelihood ratio tests (LRTs). Pairwise comparisons were done to identify statistically significant difference in adjusted geometric mean FeNO levels between categories of children who had different profiles based on their *NOS2* promoter haplotype copy number and dichotomized road length measures. Missing indicators were used to handle for missing data. All the models were adjusted for age, sex, race/ethnicity, parental education (proxy for socioeconomic status), asthma, history of respiratory allergy, and genetic ancestry to account for population stratification and community of residence as sampling design was based on communities. We performed sensitivity analyses by (a) restricting the analysis to children without asthma (87.4% of the sample), (b) restricting to children who were not exposed to secondhand tobacco smoke (95% of the sample). Because asthma status and exposure to secondhand tobacco smoke may affect FeNO, we conducted these sensitivity analyses to further investigate whether asthma status or secondhand tobacco smoke exposure could explain the observed findings. We also evaluated whether the results vary by asthma, gender, or race/ethnicity using LRTs. All tests were two-sided at a 5% significance level. All analyses were performed using the Statistical Analysis System software (SAS version 9.4; SAS Institute Inc., Cary, NC).

## Results

The average age of children was 9 years with nearly equal proportion of boys and girls (Table 1). The majority of subjects were Hispanic white (61.3%). More than half of the children (56%) had history of respiratory allergy and 12.6% of children had physician-diagnosed asthma. Age, race/ethnicity, asthma, respiratory allergy and parental education were statistically significant associated with elevated FeNO; however, sex, exposure to secondhand tobacco smoke and BMI were not significantly associated with FeNO.

**Table 1:** Selected characteristics of the study population and their bivariate relationships with FeNO^a^ (N = 2,457).

Characteristics	N^b^	%	Geometric Mean FeNO (ppb) (95%CI)]		*P*-value^c^
Age (years) [mean range] ^d^	2457	9.3 (7.3–11.5)	14.4%	(10.2% to 18.8%)	**<0.0001**
Sex					
Girls	1248	50.8	13.89	(13.41 to 14.39)	0.46
Boys	1209	49.2	13.63	(13.15 to 14.13)	
Race/Ethnicity					
Non-Hispanic white	951	38.7	13.24	(12.72 to 13.79)	**0.02**
Hispanic white	1506	61.3	14.10	(13.66 to 14.56)	
Asthma					
No	2148	87.4	13.24	(12.89 to 13.59)	**<0.0001**
Yes	309	12.6	18.06	(16.84 to 19.36)	
History of respiratory allergy					
No	1079	44.0	12.44	(11.98 to 12.91)	**<0.0001**
Yes	1376	56.0	14.91	(14.43 to 15.42)	
Exposure to secondhand smoke					
No	2122	95.0	13.64	(13.28 to 14.01)	0.99
Yes	113	5.0	13.64	(12.15 to 15.31)	
Body mass index categories					
Underweight (<5^th^ percentile)	44	1.8	13.84	(11.48 to 16.69)	0.99
Normal (5^th^-85^th^ percentile)	1505	61.5	13.79	(13.36 to 14.24)	
Overweight (85^th^ to 95^th^ percentile)	381	15.6	13.61	(12.77 to 14.50)	
Obese (≥95^th^ percentile)	517	21.1	13.73	(13.00 to 14.50)	
Parental education					
<12^th^ grade	500	21.3	14.46	(13.68 to 15.29)	**0.01**
12^th^ grade	436	18.6	13.61	(12.82 to 14.45)	
Some college	894	38.1	14.13	(13.55 to 14.75)	
College	278	11.8	13.04	(12.10 to 14.06)	
Some graduate	240	10.2	12.38	(11.42 to 13.42)	
Annual family income ($)					
<14,999	312	14.8	14.41	(13.43 to 15.47)	0.16
15,000–49,999	695	33.1	14.03	(13.38 to 14.71)	
≥49,999	1096	52.1	13.45	(12.95 to 13.97)	

^a^ Study subjects included Hispanic and Non-Hispanic white children in the Children’s Health Study who had FeNO measured in 2005–2006 and had genotypic and exposure (local road lengths around homes) data available.

^b^ Numbers do not always add up due to missing values.

^c^ *P*-values from logistic regression testing overall association of the variable with FeNO level. Statistically significant P-values are in **bold**.

^d^ Age (continuous) is presented as mean and range; percent difference in FeNO per year is presented with 95% CI.

The distributions and correlations of local road lengths around homes are presented in Table 2. Within 50m, 100m, and 200m circular buffers around subjects' home, the average road lengths were 116m, 365m, and 1344m, respectively. These traffic exposure metrics were positively correlated (P <0.0001), with stronger correlation between road lengths within 100m and 200m buffers. The two most common *NOS2* promoter haplotypes, H1 and H2, represented more than 60% of the haplotype diversity in the study population (Table 3). These haplotypes have modest negative correlation (Spearman correlation coefficient = -0.39, P <0.0001).

**Table 2:** Distributions and correlations of local road lengths.

Local road lengths within circular buffers (m)	Mean	SD	Median	25^th^ percentile	75^th^ percentile	Local road length in 50m buffer	Local road length in 100m buffer
Local road length in 50m buffer (m)	115.6	48.5	100.0	98.2	145.1		
Local road length in 100m buffer (m)	365.2	155.3	362.2	258.3	469.4	0.60 ^a^	
Local road length in 200m buffer (m)	1344.3	514.6	1380.9	1014.6	1709.3	0.42 ^a^	0.77 ^a^

^a^ P <0.0001 for Spearman correlation coefficients. SD, standard deviation

**Table 3:** Haplotype frequency of the *NOS2* promoter haplotypes.

Haplotype ^a^	Haplotype frequency	
	Non-Hispanic white	Hispanic white
h0111101 (H1)	0.35	0.31
h1000010 (H2)	0.27	0.37

^a^ SNP order in *NOS2A* promoter haplotypes is rs4795080-rs2779253-rs1889022-rs10853181-rs2531866-rs1014025-rs2531872. Within each haplotype (h), ‘0’ and ‘1’ represent the common and the variant alleles at the ordered SNP position, respectively.

**Fig 1:**
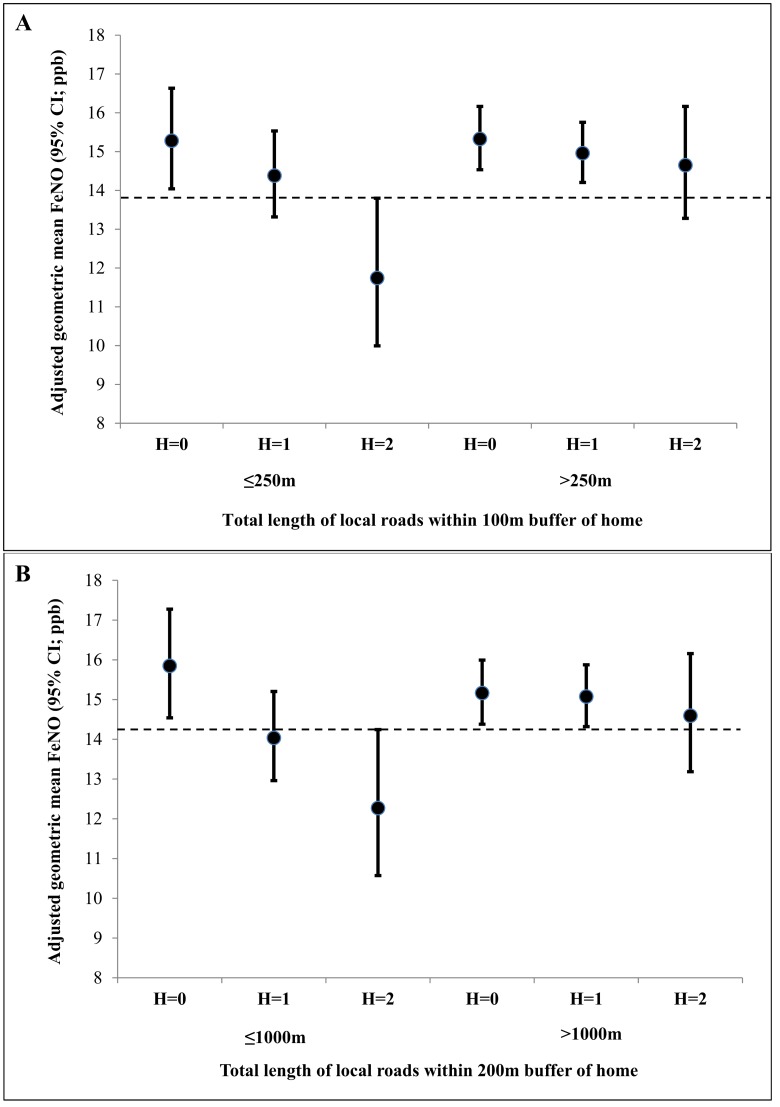
Influence of *NOS2* H1 haplotype copies on the association between length of local roads around home and FeNO. The x-axis shows H1 haplotype copies (0, 1, and 2) within categories of local road-lengths within (A) 100m and (B) 200m circular buffers of home. The circles represent adjusted geometric mean FeNO and the vertical bars represent 95% confidence intervals. The 95%CIs for the adjusted geometric means that cross the dashed horizontal line in each figure are not statistically significantly different from the adjusted geometric mean FeNO in children who had 2 copies of the H1 haplotype and had less than 250m and 1000m total local road lengths within 100m and 200m buffers, respectively.

*NOS2* H1 promoter haplotype modified the associations of length of local roads around 100m and 200m buffers and FeNO (both p-values for interaction ≤0.03; Table 4). However, we did not find any significant interactions between *NOS2* H2 promoter haplotype and any of the local road length measures around home. To further investigate the joint effects of copy number of the H1 haplotype (i.e., 0, 1 and 2) and local road lengths within 100m and 200m buffer on FeNO level, we dichotomized the road length measures by choosing a cut-point close to their 25th percentile values (i.e., 250m and 1000m for road lengths around 100m and 200m buffers, respectively). We found that children with 2 copies of the H1 haplotype who had fewer roads within 100m and 200m buffer around their homes had significantly lower FeNO compared to children who had similar exposure but no copies of the H1 haplotype (Fig 1 and Table 5). This protective effect of H1 haplotype on FeNO was abrogated in children who lived in homes surrounded by more roads (i.e. had higher road lengths). Selecting different cut-points for road lengths using quartiles provided similar results (data not shown).

**Table 4:** Influence of *NOS2* promoter haplotypes on the association between local road lengths measures and FeNO (N = 2,457).

Factor ^a^	Estimates (95% CI, ppb)^b^	Factor^a^	Estimates (95% CI; ppb)^b^
**Joint effects of H1 haplotype and road lengths within 50m buffer**		**Joint effects of H2 haplotype and road lengths within 50m buffer**	
H1	-0.043 (-0.081 to -0.006)	H2	0.017 (-0.019 to 0.052)
Local road lengths within 50m buffer	0.049 (-0.001 to 0.099)	Local road lengths within 50m buffer	0.051 (0.001 to 0.101)
H1 x Local road lengths within 50m buffer	0.062 (-0.012 to 0.137)	H2 x Local road lengths within 50m buffer	-0.001 (-0.070 to 0.071)
*P value for interaction* ^c^	0.10	*P value for interaction* ^c^	0.99
**Joint effects of H1 haplotype and road lengths within 100m buffer**		**Joint effects of H2 haplotype and road lengths within 100m buffer**	
H1	-0.043 (-0.080 to -0.005)	H2	0.017 (-0.019 to 0.052)
Local road lengths within 100m buffer	0.054 (0.006 to 0.102)	Local road lengths within 100m buffer	0.056 (0.007 to 0.104)
H1 x Local road lengths within 100m buffer	0.083 (0.012 to 0.154)	H2 x Local road lengths within 100m buffer	-0.007 (-0.074 to 0.060)
*P value for interaction* ^c^	**0.02**	*P value for interaction* ^c^	0.84
**Joint effects of H1 haplotype and road lengths within 200m buffer**		**Joint effects of H2 haplotype and road lengths within 200m buffer**	
H1	-0.042 (-0.079 to -0.004)	H2	0.017 (-0.018 to 0.053)
Local road lengths within 200m buffer	0.064 (0.012 to 0.115)	Local road lengths within 200m buffer	0.067 (0.015 to 0.118)
H1 x Local road lengths within 200m buffer	0.077 (0.006 to 0.148)	H2 x Local road lengths within 200m buffer	0.012 (-0.057 to 0.081)
*P value for interaction* ^c^	**0.03**	*P value for interaction* ^c^	0.73

^a^ Road length variables were centered at their respective mean values. The 'x' between factors represents interaction terms.

^b^ Estimates (95% confidence intervals) represent natural log transformed FeNO obtain from multivariate liner regression models associated with each factor. All models were adjusted for age, race/ethnicity, parental education, asthma, respiratory allergy, genetic ancestry and community of residence. The estimates for road lengths were scaled to 100m, 300m, and 1000m for total length of roads in 50m, 100m, and 200m buffers, respectively. The estimate for each haplotype is for per-copy of the haplotype compared to those with no copies of the respective haplotype.

^c^P-values for interaction for total length of local roads within each buffer by *NOS2* haplotypes were obtained from likelihood ratio tests from non-stratified models with appropriate interaction terms and were based on 1 degree of freedom. Statistically significant interaction P-values are in **bold**.

**Table 5:** Joint effects of *NOS2* H1 promoter haplotype and length of local road within 100m and 200m buffers on FeNO.

Number of haplotype H1copy	**Length of local road within 100m buffer around home** ^a^	
	≤ 250m	> 250m
	Adjusted Geometric Mean FeNO (95% CI; ppb) ^b^	Adjusted Geometric Mean FeNO (95% CI; ppb) ^b^
0	15.28 (14.04 to 16.63) ^c^	15.33 (14.53 to 16.16) ^c^
1	14.38 (13.32 to 15.53) ^c^	14.96 (14.20 to 15.75) ^c^
2	11.74 (9.99 to 13.80)	14.65 (13.28 to 16.16) ^c^
P-value for trend^d^	**0.002**	0.34
	**Length of local road within 200m buffer around home** ^a^	
	≤ 1000m	> 1000m
	Adjusted Geometric Mean FeNO (95% CI; ppb) ^b^	Adjusted Geometric Mean FeNO (95% CI; ppb) ^b^
0	15.85 (14.54 to 17.27) ^c^	15.16 (14.38 to 15.99) ^c^
1	14.04 (12.96 to 15.20)	15.08 (14.32 to 15.88) ^c^
2	12.27 (10.57 to 14.24)	14.59 (13.18 to 16.16)
P-value for trend^d^	**0.0005**	0.61

^a^ Road length cutoffs were selected approximately at the 25th percentile distributions.

^b^ All models were adjusted for age, race/ethnicity, parental education, asthma, respiratory allergy, genetic ancestry and community of residence.

^c^ Adjusted geometric mean FeNO is significantly different compared to the adjusted geometric mean FeNO level in children who had 2 copies of the H1 haplotype and had less than 250m and 1000m total local road lengths within 100m and 200m buffers, respectively.

^d^ P-values for trend were obtained from stratified models within each categories of road lengths and were obtained from treating the haplotype copy numbers as continuous variables. These models were adjusted for age, race/ethnicity, parental education, asthma, respiratory allergy, genetic ancestry and community of residence. Statistically significant P-values for trend are in **bold**.

We evaluated the joint effects of asthma, *NOS2* H1 promoter haplotype and length of local roads around home on FeNO in children. While children with asthma had significantly higher FeNO than those without asthma, there were no statistically significant three-way interactive effects of asthma, *NOS2* H1 haplotype and road lengths within 100m and 200m buffers on FeNO (both P-values for interaction > 0.6; S1 Table). Restricting the analysis to children without asthma (87.4% of the sample) or those who were not exposed to secondhand tobacco smoke (95% of the sample) did not affect the observed findings (data not shown). Furthermore, the joint effect the *NOS2* H1 promoter haplotype and length of local roads on FeNO did not vary by gender, race/ethnicity (data not shown).

## Discussion

We found that one of the most common *NOS2* promoter haplotypes and length of local road within 100m and 200m buffers jointly influence FeNO level in children. Our results show that the protective effect of the promoter haplotype on FeNO was evident in children who had fewer roads around their homes. However, in children who lived in homes surrounded by more roads, the protective effect of the haplotype was lost. This novel finding provides evidence that joint evaluation of DNA sequence variants in *NOS2* and traffic-related exposure is needed to understand interindividual differences in FeNO levels in children.

Using data from the CHS cohort, we previously reported that local road length within 100m and 200m buffers were significantly associated with higher FeNO [12], and per copy of *NOS2* promoter haplotype H1 was significantly associated with 6.2% lower FeNO [15]. In this paper, we extend these findings by showing joint effects of genetic and environmental exposures on FeNO. We speculate that in children carrying two copies of the H1 haplotype, the protective effects of the haplotype on FeNO is lost due to higher traffic-related exposure, as the latter may induce iNOS enzymatic activity [30, 31]. Although the tagSNPs were able to characterize the haplotype diversity of the *NOS2* promoter region, more research is warranted to identify the causal variants that may mediate these effects on FeNO. Furthermore, experimental studies are required to evaluate iNOS enzyme activity in relation to the DNA sequence variations in *NOS2* promoter and traffic-related exposures.

To limit the number of hypothesis tests, we restricted our testing of gene-environment interaction by investigating the joint effects of two of the most common promoter haplotypes of *NOS2* (the major determinant of NO in airway) [11], and a set of three traffic measures that have been previously found to be associated with FeNO [12, 21, 22]. Of the six tests we conducted for joint effects (Table 4), two were statistically significant suggesting that the observed results are less likely due to chance.

We used one of the surrogates for traffic-related exposure that has been consistently associated with higher FeNO in earlier studies, while other traffic metrics (e.g., modeled total oxides of nitrogen [NOx], traffic density, distance to freeway and major roads) did not show significant associations with FeNO in our previous analysis [12]. It is currently unknown which specific source of traffic-related exposure (fresh gasoline/diesel exhaust, brake wear metals, re-suspended road dust, etc) underlie the observed relationships. While most studies conducted to date mostly focused on relating GIS based traffic metrics as predictors for NO2 or particular matter pollutants using land use regression models, there is paucity of studies that attempted to disentangle how each metrics may capture different exposure signals. It is plausible that road length around home captures exposure from specific noxious agent(s) whereas others traffic exposure metrics may not. Near-road traffic emissions can be complexly impacted by many factors, such as temporal and spatial patterns of traffic activity and meteorology [32]. It could be also impacted by exposures related to acceleration and brake wear due to traffic stop signs in residential areas [33]. Further research is warranted to identify specific exposures from near-roadway pollution that may underlie the direct and interactive effects of the surrogate exposures on FeNO. It is also interesting to note that traffic count data in local roads are often unavailable from the state or city departments of transportation while such data from freeways and major roads are often available, which may have limited estimation of near-home traffic-related exposure from existing exposure modeling approaches. Further research is needed to better understand the local sources of residential-level traffic-related exposures so that better exposure estimation modeling approach could be utilized to capture short- and long-term traffic related exposures near homes.

Our use of buccal mucosal cells does not allow relating the haplotypes to gene expression and normal buccal epithelial cells do not express iNOS. Collection of bronchial epithelial cells (which express iNOS) from a large sample of children who were recruited from public school classrooms was not feasible due to risks associated with such invasive testing. In addition, *NOS2* is the main determinant of FeNO in airway, and different cell types in lungs (airway epithelium, inflammatory cells such as macrophages, neutrophils and mast cells) produce NO [9]. Therefore, our measurement of FeNO was not only feasible in a large number of school children it also may have provided a valid proxy for the *in vivo* iNOS expression from these different cell types. Previously, we reported that *NOS2* promoter haplotypes affect FeNO level [15] which provided some support for the biological significance of the genetic variants under study.

Data on animal models on biological effects of traffic-related air pollutants on airways in animals that are deficient in iNOS (i.e., *NOS2* knockout) or in animals undergoing iNOS inhibition have been very limited. In one study conducted in mice that were chronically exposed to tobacco smoke, iNOS deficient mice were protected from developing emphysema [34]. In addition, in wild type mice, iNOS inhibition prior to tobacco smoke exposure prevented development of emphysema and reversed emphysematous changes that developed emphysema following chronic tobacco smoke exposure [34]. These findings suggest that excessive NO synthesis in airway due to air pollutants has detrimental effects. Our current finding of a joint effect of traffic-related air pollution and *NOS2* promoter haplotypes on FeNO provides one mechanism through which air pollutants could modulate nitrosative stress in children’s airways.

The strengths of the study include relative large sample size from a well-characterized population-based study. Children living in 12 Southern California communities had a wide contrast in local road length measures. We also minimized population stratification by adjusting for genetic ancestry in all our models. However, interpretation of our results requires the consideration of some study limitations. We used parental report to define the health outcomes, and concern has been raised that parental report might not reflect physician diagnosis. Based on medical records review, we have previously found strong evidence that parental report reflected physician diagnosis [35]. Although asthma prevalence rate in our study population are in line with national prevalence estimates, the study was not adequately powered to detect significant three-way interactive effects of asthma, *NOS2* haplotype and road length measures. Genotyping data were only available on Hispanic and non-Hispanic white subjects, so the study findings may not be generalizable to other ethnic groups.

## Conclusions

We found that the protective role of a common *NOS2* promoter haplotype on FeNO level in children was lost when children’s lived near busy traffic. Because FeNO is a biomarker of airway inflammation, our findings suggest that at high level of traffic-related exposure, children may not benefit from having a protective DNA sequence variants in *NOS2* promoter. Because the *NOS2* promoter haplotype frequency that showed joint effects with traffic exposure for FeNO is quite common, our findings may have implications for public health and transportation planning in residential areas so that traffic-related exposures could be reduced to protect children’s respiratory health.

## Supporting Information

S1 Table Joint effects of asthma, *NOS2* promoter haplotype H1 and road length measures on FeNO. (DOCX)
