# Correction: Inducible Nitric Oxide Synthase Promoter Haplotypes and Residential Traffic-Related Air Pollution Jointly Influence Exhaled Nitric Oxide Level in Children

**DOI:** 10.1371/journal.pone.0155567

**Published:** 2016-05-09

**Authors:** Muhammad T. Salam, Pi-Chu Lin, Sandrah P. Eckel, W. James Gauderman, Frank D. Gilliland

There is an error in footnote (c) of Table 1. The correct sentence is: P-values from linear regression testing overall association of the variable with natural log-transformed FeNO level. Statistically significant P-values are in **bold**.

**Table 1:** Selected characteristics of the study population and their bivariate relationships with FeNO^a^ (N = 2,457).

Characteristics	N^b^	%	Geometric Mean FeNO (ppb) (95%CI)]		*P*-value^c^
Age (years) [mean range] ^d^	2457	9.3 (7.3–11.5)	14.4%	(10.2% to 18.8%)	**<0.0001**
Sex					
Girls	1248	50.8	13.89	(13.41 to 14.39)	0.46
Boys	1209	49.2	13.63	(13.15 to 14.13)	
Race/Ethnicity					
Non-Hispanic white	951	38.7	13.24	(12.72 to 13.79)	**0.02**
Hispanic white	1506	61.3	14.10	(13.66 to 14.56)	
Asthma					
No	2148	87.4	13.24	(12.89 to 13.59)	**<0.0001**
Yes	309	12.6	18.06	(16.84 to 19.36)	
History of respiratory allergy					
No	1079	44.0	12.44	(11.98 to 12.91)	**<0.0001**
Yes	1376	56.0	14.91	(14.43 to 15.42)	
Exposure to secondhand smoke					
No	2122	95.0	13.64	(13.28 to 14.01)	0.99
Yes	113	5.0	13.64	(12.15 to 15.31)	
Body mass index categories					
Underweight (<5^th^ percentile)	44	1.8	13.84	(11.48 to 16.69)	0.99
Normal (5^th^-85^th^ percentile)	1505	61.5	13.79	(13.36 to 14.24)	
Overweight (85^th^ to 95^th^ percentile)	381	15.6	13.61	(12.77 to 14.50)	
Obese (≥95^th^ percentile)	517	21.1	13.73	(13.00 to 14.50)	
Parental education					
<12^th^ grade	500	21.3	14.46	(13.68 to 15.29)	**0.01**
12^th^ grade	436	18.6	13.61	(12.82 to 14.45)	
Some college	894	38.1	14.13	(13.55 to 14.75)	
College	278	11.8	13.04	(12.10 to 14.06)	
Some graduate	240	10.2	12.38	(11.42 to 13.42)	
Annual family income ($)					
<14,999	312	14.8	14.41	(13.43 to 15.47)	0.16
15,000–49,999	695	33.1	14.03	(13.38 to 14.71)	
≥49,999	1096	52.1	13.45	(12.95 to 13.97)	

^a^ Study subjects included Hispanic and Non-Hispanic white children in the Children’s Health Study who had FeNO measured in 2005–2006 and had genotypic and exposure (local road lengths around homes) data available.

^b^ Numbers do not always add up due to missing values.

^c^ *P*-values from linear regression testing overall association of the variable with natural log-transformed FeNO level. Statistically significant P-values are in **bold**.

^d^ Age (continuous) is presented as mean and range; percent difference in FeNO per year is presented with 95% CI.
