# Supplementary material for: Inducible Nitric Oxide Synthase Promoter Haplotypes and Residential Traffic-Related Air Pollution Jointly Influence Exhaled Nitric Oxide Level in Children
Source: PLoS One. 2015 Dec 29;10(12):e0145363. doi: 10.1371/journal.pone.0145363 (PMC4695093; doi:10.1371/journal.pone.0145363)
Supplement: S1 Table — (DOCX) [file pone.0145363.s001.docx]

**S1 Table. Joint effects of asthma, *NOS2* promoter haplotype H1 and road length measures on FeNO**

| Factors^a^ | Estimates (95% CI, ppb) ^b^ | P value^c^ |
| --- | --- | --- |
| **Length of local road within 100-m buffer around home** |  |  |
| H1 | -0.03 (-0.07 to 0.01) | 0.10 |
| Local road lengths within 100m buffer | 0.04 (-0.01 to 0.09) | 0.12 |
| Asthma | 0.31 (0.24 to 0.39) | <0.0001 |
| H1x Local road lengths within 100m buffer | 0.08 (0.00 to 0.16) | 0.05 |
| H1x Asthma | -0.12 (-0.23 to -0.00) | 0.05 |
| Asthma x Local road lengths within 100m buffer | 0.11 (-0.02 to 0.25) | 0.10 |
| Asthma x H1 x Local road lengths within 100m buffer | -0.03 (-0.22 to 0.17) | 0.79 |
|  |  |  |
| **Length of local road within 200-m buffer around home** |  |  |
| H1 | -0.03 (-0.07 to 0.01) | 0.12 |
| Local road lengths within 200m buffer | 0.06 (0.01 to 0.12) | 0.02 |
| Asthma | 0.31 (0.24 to 0.39) | <0.0001 |
| H1x Local road lengths within 200m buffer | 0.08 (0.00 to 0.16) | 0.04 |
| H1x Asthma | -0.12 (-0.23 to 0.00) | 0.05 |
| Asthma x Local road lengths within 200m buffer | -0.00 (-0.14 to 0.14) | 0.96 |
| Asthma x H1 x Local road lengths within 200m buffer | -0.04 (-0.25 to 0.17) | 0.69 |

^a^ Road length variables were centered at their respective mean values. The 'x' between factors represents interaction terms.

^b^Estimates (95% confidence intervals) represent natural log transformed FeNO associated with each factor. All models were adjusted for race/ethnicity, ancestry and community of residence. The estimates for road lengths were scaled to 300m, and 1000m for total length of roads in 100m, and 200m buffers, respectively.

^c^ P-values for the association of each of the main effects and interaction terms with FeNO.
